# Multi-institutional analysis of cervical esophageal carcinoma patients treated with definitive chemoradiotherapy: TROD 01-005 study

**DOI:** 10.32604/or.2023.028840

**Published:** 2023-05-24

**Authors:** OZAN CEM GULER, EZGI OYMAK, GOZDE YAZICI, OZLEM OZKAYA AKAGUNDUZ, OGUZ CETINAYAK, PETEK ERPOLAT, ATIL AKSOY, MURSEL DUZOVA, BERNA AKKUS YILDIRIM, MERAL KURT, EMINE CANYILMAZ, GULER YAVAS, SERAP AKYUREK, DIDEM COLPAN OKSUZ, ESRA KAYTAN SAGLAM, OMUR KARAKOYUN CELIK, ENIS OZYAR, MUSTAFA CENGIZ, CEM ONAL

**Affiliations:** 1 Department of Radiation Oncology, Faculty of Medicine, Baskent University, Adana Dr. Turgut Noyan Research and Treatment Center, Adana, Turkey; 2 Radiation Oncology Unit, Iskenderun Gelisim Hospital, Hatay, Turkey; 3 Department of Radiation Oncology, Faculty of Medicine, Hacettepe University, Ankara, Turkey; 4 Department of Radiation Oncology, Faculty of Medicine, Ege University, Izmir, Turkey; 5 Department of Radiation Oncology, Faculty of Medicine, 9 Eylul University, Izmir, Turkey; 6 Department of Radiation Oncology, Faculty of Medicine, Gazi University, Ankara, Turkey; 7 Department of Radiation Oncology, Faculty of Medicine, Akdeniz University, Antalya, Turkey; 8 Department of Radiation Oncology, Faculty of Medicine, Selcuk University, Konya, Turkey; 9 Radiation Oncology Unit, Prof. Dr. Cemil Tascioglu Hospital, İstanbul, Turkey; 10 Department of Radiation Oncology, Faculty of Medicine, Uludag University, Bursa, Turkey; 11 Department of Radiation Oncology, Faculty of Medicine, Karadeniz Technical University, Trabzon, Turkey; 12 Department of Radiation Oncology, Faculty of Medicine, Baskent University, Ankara, Turkey; 13 Department of Radiation Oncology, Faculty of Medicine, Ankara University, Ankara, Turkey; 14 Department of Radiation Oncology, Faculty of Medicine, Istanbul University Cerrahpasa, Istanbul, Turkey; 15 Radiation Oncology Unit, Memorial Sisli Hospital, Istanbul, Turkey; 16 Department of Radiation Oncology, Faculty of Medicine, Celal Bayar University, Manisa, Turkey; 17 Department of Radiation Oncology, Acibadem University Maslak Hospital, Istanbul, Turkey

**Keywords:** Esophageal cancer, Chemoradiotherapy, Radiotherapy, Local control, Toxicity, Survival

## Abstract

The aim of this study was to examine the prognostic factors and treatment outcomes of cervical esophageal carcinoma (CEC) patients who underwent definitive chemoradiotherapy (CRT). The clinical data of 175 biopsy-confirmed CEC patients treated with definitive CRT between April 2005 and September 2021 were retrospectively analyzed. The prognostic factors predicting overall survival (OS), progression-free survival (PFS), and local recurrence-free survival (LRFS) were assessed in uni- and multivariable analyses. The median age of the entire cohort was 56 years (range: 26–87 years). All patients received definitive radiotherapy with a median total dose of 60 Gy, and 52% of the patients received cisplatin-based concurrent chemotherapy. The 2-year OS, PFS, and LRFS rates were 58.8%, 46.9%, and 52.4%, respectively, with a median follow-up duration of 41.6 months. Patients’ performance status, clinical nodal stage, tumor size, and treatment response were significant prognostic factors for OS, PFS, and LRFS in univariate analysis. Non-complete treatment response was an independent predictor for poor OS (HR = 4.41, 95% CI, 2.78–7.00, *p* < 0.001) and PFS (HR = 4.28, 95% CI, 2.79–6.58, *p* < 0.001), whereas poor performance score was a predictor for worse LRFS (HR = 1.83, 95% CI, 1.12–2.98, *p* = 0.02) in multivariable analysis. Fifty-two patients (29.7%) experienced grade II or higher toxicity. In this multicenter study, we demonstrated that definitive CRT is a safe and effective treatment for patients with CEC. Higher radiation doses were found to have no effect on treatment outcomes, but a better response to treatment and a better patient performance status did.

## Introduction

Esophageal cancer (EC) is the seventh most common type of cancer and the sixth leading cause of cancer death worldwide [1]. Cervical esophageal carcinoma (CEC) accounts for less than 10% of esophageal tumors, and most patients have locally advanced disease when they are first diagnosed [2,3]. The management of CEC is challenging because it is frequently associated with head and neck cancer rather than the middle or lower third of esophageal adenocarcinoma [3]. Surgical management of CEC with pharyngo-laryngo-esophagectomy through cervical, abdominal, and thoracic incisions and a permanent terminal tracheostomy is difficult due to high morbidity and mortality rates attributable to the close proximity of the larynx, trachea, and major vascular structures [3]. According to National Comprehensive Cancer Network (NCCN) and European Society for Medical Oncology (ESMO) guidelines, definitive chemoradiotherapy (CRT) is the standard treatment modality in the management of CEC [4,5].

Previous research has demonstrated that primary tumor control in CEC patients is a surrogate for improved survival, and that patients with a good local treatment response fared better than non-responders [6]. Despite advances in radiotherapy (RT) delivery methods and novel systemic chemotherapy agents, the outcomes of patients with CEC remain poor, with a median overall survival time of 33 months; this may be due to the unpredictability of high rates of local failure (LF). As a result of the low incidence of disease and the lack of prospective studies, evidence regarding the optimal irradiation technique and radiation doses is lacking. In addition, no randomized clinical trials supporting dose escalation in EC patients have been performed [7,8].

Based on these findings, we conducted a multicentric study investigating the treatment outcomes of CEC patients treated with definitive CRT. Additionally, prognostic factors for overall survival (OS), progression-free survival (PFS), and local control (LC) were assessed.

## Materials and Methods

### Patient selection

Between April 2005 and September 2021, 175 biopsy-confirmed CEC patients who received definitive CRT at 17 national institutions were analyzed retrospectively. Patients with SCC histology, receiving at least one cycle of concurrent chemotherapy with RT, and receiving treatment with three-dimensional conformal RT (3DCRT) or intensity-modulated RT (IMRT) met the inclusion criteria. Exclusion criteria included patients with poor performance status (ECOG > 2), those having undergone surgery before CRT, those with distant metastases, and those treated with palliative intent. To prevent the inclusion of hypopharyngeal cancer, patients were excluded if the tumor extended cranially beyond the level of the hyoid bone.

This retrospective study complied with the regulations of the principles of Helsinki. This study protocol was reviewed and approved by the Baskent University Review Board, approval number (KA22/55).

### Treatment characteristics

The treatment protocol included concurrent chemotherapy (cisplatin alone, cisplatin and 5-FU, carbo/taxol, or others) and RT. Adjuvant chemotherapy or induction chemotherapy was administered according to the decision of the treating physician. The decision to perform percutaneous endoscopic gastrostomy on a patient was based on the patient’s performance and nutritional status.

For RT planning, a multi-slice planning computed tomography (CT) with thermoplastic mask was used for all patients. For better delineation of the primary tumor and lymph nodes, magnetic resonance imaging (MRI) and/or positron emission tomography CT (PET-CT) were fused with the planning CT at the time of initial diagnosis. On planning CT and fusion images, the gross tumor volume (GTV) comprises the visible primary tumor and pathological lymph nodes. Clinical tumor volume (CTV) includes pathological lymph nodes with a margin of 5–10 mm and the primary tumor with a margin of 1–2 cm. Due to each institution’s RT technique and protocol, planning tumor volume (PTV) comprised CTV with adequate margins. Regional lymph node irradiation or elective nodal irradiation (ENI) was performed on each patient depending on the decision of the treating physician.

### Follow-up

Patients were seen every three months for the first two years, every six months between three and five years, and annually thereafter or more frequently if necessary. Every visit included a thorough physical examination that included an endoscopic examination. Toxicities were gathered from an institutional database and reported using the Common Terminology Criteria for Adverse Events, version 4.0.

For treatment response assessment, the initial imaging modality was repeated. For metabolic response evaluation, Positron Emission Tomography Response Criteria in Solid Tumors (PERCIST), version 1.0, was used [9]. The RECIST version 1.1 classification system was used to categorize the radiologic responses that were collected during the initial radiological assessment [10]. These responses were categorized as a complete response (CR), a partial response (PR), stable disease (SD), or progression of disease (PD).

### Statistical analysis

For statistical analysis, we utilized SPSS 22.0 (SPSS for Windows, IBM Corp., Armonk, NY, USA) and GraphPad Prism version 9.3.1 (GraphPad Software Inc., San Diego, USA). When comparing clinical and pathological factors between patients, Chi-square ( $\chi^{2}$ ) and Student’s *t*-tests were utilized as statistical tools. The progression-free survival (PFS) was measured as the period of time that elapsed between the last date of CRT and the date of radiological detection of progression of the target volume or distant metastasis following CRT. This measurement was based on whichever came first: the progression of the target volume or the distant metastasis. The time until death was determined by taking the interval of time that passed between the completion of the CRT and the time of the last follow-up. In order to carry out univariate analysis, the log-rank test was utilized. The Cox proportional hazards model and any covariates that had a *p* value of less than 0.05 in the univariate analysis were included in the multivariate analyses that were carried out. A *p* value of less than 0.05 was considered statistically significant.

## Results

### Patient characteristics

Data from 175 patients were analyzed. Patients and treatment characteristics are summarized in Table 1. The majority of patients were female (59.4%), had an advanced clinical T stage (77.7%), had good performance status (81.7%), and had regional lymph node metastasis (62.3%). Most of the patients (73.7%) were treated between 2014 and 2021. The majority of patients (80.5%) were staged using PET-CT. A total of 114 patients (65.1%) had PEG before treatment.

**Table 1:** Patient and treatment characteristics

Median age (years, range)	56 (26–87)
Sex (n, %)	
Male	71 (40.6)
Female	104 (59.4)
ECOG	
0	59 (33.7)
I	84 (48.0)
II	32 (18.3)
T stage	
T1	3 (1.7)
T2	36 (20.6)
T3	77 (44.0)
T4	59 (33.7)
N stage	
N0	66 (37.7)
N1	52 (29.7)
N2	46 (26.3)
N3	11 (6.3)
Staging	
BT	22 (12.6)
MRI	12 (6.9)
PET	141 (80.5)
RT technique	
3D-CRT	30 (17.1)
IMRT	145 (82.9)
RT dose	
Fraction dose	2 Gy (1.8–2.3 Gy)
Fraction number	30 (23–38)
Total dose	60 Gy (45–72.6)
Concurrent chemotherapy (n, %)	
Cisplatin	88 (50)
Carbo/taxol	68 (39)
Others	19 (11)

Abbreviations: CRT: chemoradiotherapy, CT: computed tomography, IMRT: intensity modulated radiotherapy, MRI: magnetic resonance imaging, PET: positron emission tomography, RT: radiotherapy.

The median fraction and total radiation doses were 2 Gy (range: 1.8–2.3 Gy) and 60 Gy (range: 45–72 Gy), respectively, and were delivered in a median of 30 fractions (range: 23–38). The median BED10 was 72 Gy (range: 53.1–88.5). Thirty patients (17.1%) were treated with 3-D conformal RT, and 145 patients (82.9%) received intensity-modulated RT (IMRT). Half of the patients (50%) received concurrent cisplatin-based chemotherapy with a median of 5 cycles (range: 1–8). Twenty-three patients had concurrent cisplatin and 5FU, while 63 patients received only cisplatin concurrent with RT.

### Treatment outcomes

**Figure 1:**
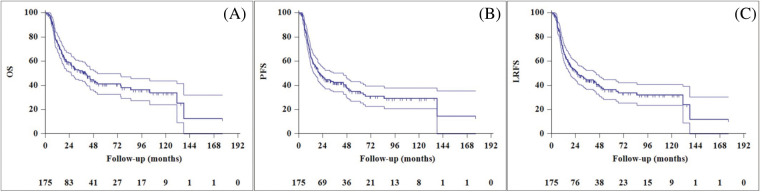
Overall survival (A), progression-free survival (B), and local recurrence free survival (C) curves of entire cohort.

The median follow-up time for the entire cohort was 41.6 months (95% CI, 26.2–57 months). The two-year OS, PFS, and local recurrence-free survival (LRFS) rates were 58.8%, 46.9%, and 52.4%, respectively (Fig. 1). At the last visit, 83 patients (47.4%) were alive and 92 patients (52.6%) had died. Progression occurred in 76 patients (43.4%) at a median of 11.6 months (range: 3.6–85.3 months) after completion of treatment. Of the 76 patients with progression, 37 (21.1%) had local recurrence (LR), 21 (12%) had distant metastasis (DM), and 18 (10.3%) had both LR and DM.

### Prognostic factors

The median OS duration was 40.1 months (95% CI, 27.5–52.7). Univariate analysis revealed that performance status, tumor size, clinical nodal stage, and treatment response were significant prognostic factors for OS (Table 2). Multivariable analysis found inadequate treatment response to be the only predictor of shorter OS (Table 3). Multivariate analysis determined good performance status to be predictive of improved OS, and this was close to being statistically significant (*p* = 0.06). Univariate analysis found performance status, tumor size, nodal stage, and treatment response to be significant prognostic factors for predicting PFS. Patients with non-CR had inferior PFS, as demonstrated by multivariable analysis, and poor performance status was predictive of worse PFS at a level close to statistical significance.

**Table 2:** Univariate analysis for overall survival, progression-free survival, and local recurrence free survival

Covariate	n	OS HR	*p*	PFS HR	*p*	LRFS HR	*p*
		(95% CI)		(95% CI)		(95% CI)	
Sex							
Male	71	1	0.39	1	0.34	1	0.85
Female	104	1.20 (0.79–1.82)		0.83 (0.56–1.22)		0.96 (0.65–1.44)	
Age							
<60 y	100	1	0.6	1	0.61	1	0.98
≥60 y	74	0.90 (0.59–1.36)		1.11 (0.75–1.63)		0.98 (0.67–1.49)	
ECOG PS							
0-1	143	1	0.01	1	0.02	1	0.006
2	32	1.84 (1.15–2.96)		1.64 (1.07–2.52)		1.86 (1.19–2.92)	
T							
T1-2	39	1	0.62	1	0.54	1	0.56
T3-4	116	1.14 (0.68–1.89)		1.16 (0.72–1.88)		1.16 (0.71–1.84)	
N							
N0-1	118	1	0.003	1	0.002	1	0.002
N2-3	57	1.87 (1.24–2.83)		1.86 (1.26–2.74)		1.87 (1.26–2.77)	
Tumor size							
≤5 cm	116	1	0.02	1	0.04	1	0.03
>5 cm	59	1.63 (1.07–2.50)		1.53 (1.03–2.27)		1.59 (1.06–2.38)	
RT technique							
3D-conformal	30	1	0.84	1	0.88	1	0.83
IMRT	145	0.95 (0.58–1.56)		1.04 (0.64–1.68)		1.05 (0.65–1.72)	
RT dose							
≤54 Gy	73	1	0.3	1	0.1	1	0.25
>54 Gy	102	0.81 (0.53–1.22)		0.72 (0.49–1.06)		0.8 (0.54–1.18)	
Treatment period							
2005–2013	46	1	0.93	1	0.46	1	0.88
2014–2021	129	1.02 (0.63–1.67)		0.84 (0.52–1.35)		0.97 (0.63–1.48)	
Tx response							
CR	96	1	<0.001	1	<0.001	1	<0.001
Non-CR	73	4.8 (3.06–7.56)		4.54 (3.00–6.89)		5.30 (3.43–8.20)	
Conc. ChT							
Cisplatin	89	1	0.56	1	0.87	1	0.58
Others	86	0.88 (0.58–1.34)		0.87 (0.59–1.28)		0.90 (0.60–1.33)	

Abbreviations: ChT: chemotherapy, CI: confidence interval, Conc.: concurrent, CR: complete response, HR: hazard ratio, ECOG PS: Eastern Cooperative Oncology Group performance score, IMRT: intensity modulated radiotherapy, LRFS: local recurrence free survival, OS: overall survival, PFS: progression-free survival, RT: radiotherapy, Tx: treatment.

**Table 3:** Multivariate analysis for overall survival, progression-free survival, and local recurrence free survival

Variables	Risk factors	HR (95% CI)	*p*
*Overall survival*			
ECOG	0 *vs*. 1–2	0.65 (0.41–1.01)	0.06
N stage	N0–1 *vs*. N2–3	1.28 (0.83–1.97)	0.27
Tumor size	≤5 cm *vs.* >5 cm	1.16 (0.74–1.83)	0.52
Treatment response	CR *vs*. Non-CR	4.41 (2.78–7.00)	<0.001
*Progression-free survival*			
ECOG	0 *vs*. 1–2	1.54 (0.96–2.45)	0.07
N stage	N0–1 *vs*. N2–3	1.18 (0.78–1.80)	0.43
Tumor size	≤5 cm *vs.* >5 cm	1.17 (0.76–1.78)	0.48
Treatment response	CR *vs*. Non-CR	4.28 (2.79–6.58)	<0.001
*Local recurrence free survival*			
ECOG	0 *vs*. 1–2	1.83 (1.12–2.98)	0.02
N stage	N0–1 *vs*. N2–3	1.14 (0.75–1.73)	0.54
Tumor size	≤5 cm *vs.* >5 cm	1.13 (0.73–1.74)	0.58
Treatment response	CR *vs*. Non-CR	5.04 (3.22–7.89)	<0.001

**Figure 2:**
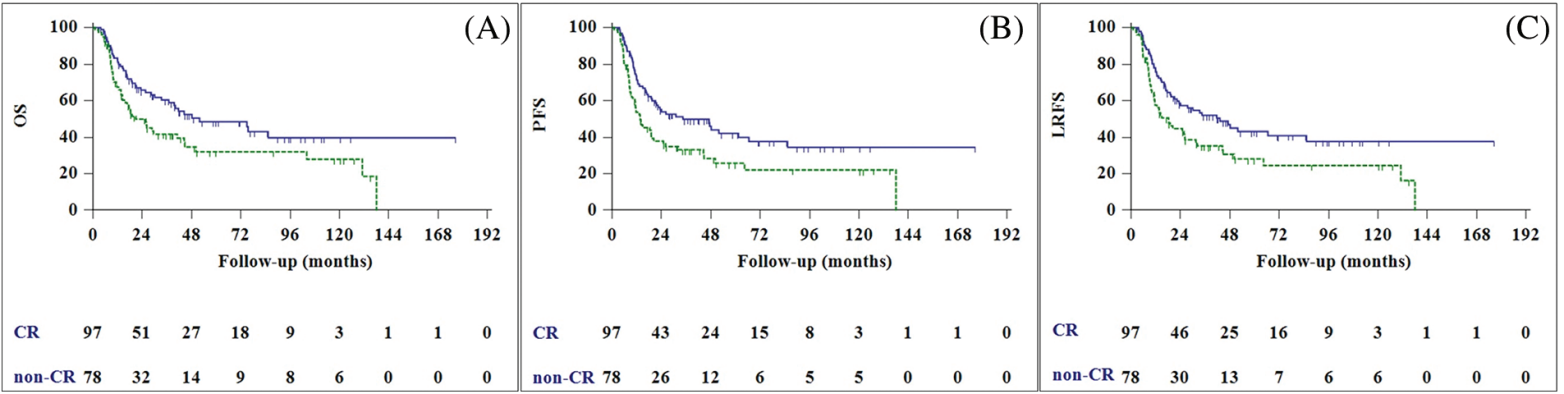
Overall survival (A), progression-free survival (B), and local recurrence free survival (C) curves of complete responder (CR) patients (blue line), and non-CR patients (green line).

Locoregional recurrence was observed in 55 patients (31.4%), and the median LRFS was 26.0 months (95% CI, 14.9–37.1 months). Univariate analysis revealed patient performance status, tumor size, nodal stage, and treatment response to be significant prognostic factors for predicting LRFS. In multivariable analysis, good performance status and complete treatment response were found to be predictors of improved LRFS (Fig. 2).

When comparing patient outcomes based on the current standard RT dose of 54 Gy (≤54 Gy *vs*. >54 Gy), no statistically significant difference in LC was found (*p* = 0.25). In addition, no statistically significant difference was seen in survival or LC between patients who received ≤60 Gy or >60 Gy. We also examined the patients by grouping them based on their median BED10 dose of 72 Gy; again, no statistically significant difference was found between patients who received BED10 ≤72 Gy and those who received BED10 >72 Gy.

### Toxicities

Fifty-two patients (29.7%) experienced grade II or higher toxicity. Twenty-five patients (14.2%) experienced grade II toxicity (22 patients with stricture and three patients with pneumonia), 23 patients (13.1%) experienced grade III toxicity (19 patients with stricture, three patients with pneumonia, and one patient with fistula), and four patients (2.3%) experienced grade IV toxicity (one patient with pneumonia, one with stricture, and two with fistula). None of the patients had myelitis.

In the high dose RT (≥54 Gy) arm, the rate of late grade II toxicities was significantly higher than in the low dose RT (54 Gy) arm (8.2% *vs*. 20.6; *p* = 0.03). When comparing late toxicities according to the chemotherapy regimens the patient received, we found no difference between the cisplatin (16.2%), cisplatin-5FU (15.0%), and carbo-taxol (14.9%) groups (*p* = 0.97).

## Discussion

Our multi-institutional retrospective analysis revealed that CRT is a safe and effective treatment for patients with CEC. In multivariate analysis, the local control of the primary tumor was the only independent predictor of survival. Higher radiation doses were found to have no effect on LC and survival at the expense of increased toxicity.

Due to the rarity of the disease, published data and treatment algorithms for CEC patients are still immature. However, current NCCN and ESMO guidelines for the treatment of CEC recommend concurrent CRT as the standard of care. [4,5,11,12]. The widely differing irradiation doses cited in the published literature, ranging from 50 to 70 Gy, contribute to the ongoing debate over the optimal dose for treating CEC [6,13–18] (Table 4). The irradiation methods and treatment modalities used in the cases discussed in the existing literature are also highly variable. Although LC rates in older series with RT alone were reported to be 25%, more recent series with improved RT techniques and dose escalation led to better outcomes [19]. Despite the fact that the highest rates of LC and CR can be achieved with concurrent CRT, the association between higher RT doses and improved primary LC in the presence of concurrent chemotherapy is debatable. However, according to the most recent NCCN guidelines for EC, higher doses of RT are recommended for patients with cervical tumors [4]. Cao et al. [20] reported a 2-year LRFS rate of 68% in their cohort of CEC patients treated with a median of 68 Gy RT alone. Zhang et al. [21] retrospectively investigated stages II–III unresectable-EC patients treated with concurrent chemotherapy and RT with a dose 51 Gy (low dose) *vs*. >51 Gy (high dose) and found that a higher radiation dose was associated with an increase in LC and an improvement in OS. Hermann et al. [22] conducted a Swiss multicenter study with 55 patients and found that those who received a total radiation dose of 56 Gy or more had significantly better outcomes compared to those receiving less than 56 Gy. Similarly, Kim et al. [23] reported that the LC of patients receiving high-dose RT with a total dose greater than 59.4 Gy was superior to that of patients receiving less than 59.4 Gy. These studies showing the benefits of higher doses of RT are not the only ones, and others have reported negative findings [15,16,18]. Huang et al. [16] investigated purely CEC patients receiving 54 Gy in 20 fractions *vs*. 70 Gy in 35 fractions with concurrent chemotherapy and concluded that higher doses of RT, prophylactic nodal irradiation, and high-dose cisplatin chemotherapy did not result in improved survival. Zhang et al. [18] found no statistically significant difference in treatment outcomes between radiation doses of ≤60 Gy and >60 Gy. Gkika et al. [15] also failed to find an advantage to dose escalation in CEC patients receiving CRT. A study using the National Cancer Database found no statistically significant difference in OS rates between standard (50–54 Gy), medium (50.4–66 Gy), and high (66–74 Gy) radiation dose groups in a total of 789 patients [24]. High-dose RT was not associated with improved treatment outcomes in our study, but it was associated with higher rates of grade II toxicities, which is consistent with the findings of the vast majority of other studies [15,16,18,24].

**Table 4:** Published studies evaluating cervical esophageal cancer patients treated with definitive chemoradiotherapy

Author, year	Design	n	Localization	ChT	RT Dose	f/u	LC	Survival	Tx
Burmeister et al. [13], 2000	R	34	CEC	CF	61.2 Gy	55 mo	–	5y OS 55%	Grade V 6%
Wang et al. [17], 2006	R	35	CEC/TEC	CF	50.4 Gy	39 mo	5y LRPFS 47.7%	5y OS 18.6%, DFS 22.4%	–
Huang et al. [16], 2008	R	50	CEC	CF	54-70 Gy	39 mo	2y LRPFS 47%	5y OS 28%	Grade III 38%
Gkika et al. [15], 2014	R	55	CEC	C	60 Gy	146 mo	5y LRPFS 47%	5y OS 25%	Grades II–III 22%
Zhang et al. [18], 2015	R	102	CEC	CF	50-70 Gy	47 mo	3y LRPFS 35.3%	3y OS 39.3%, PFS 33.6%	Grade III 24.5%
Cao et al. [14,20], 2015	R	35	CEC	C	64 Gy	17 mo	2y LFFS 68.3%	2y OS 47.6%	Grade III 10.4%
Zenda et al. [6], 2016	P	30	CEC	CF	60 Gy	41 mo	3y LFFS 52.5%	3y OS 66.5%, PFS 36.6%	Grade III 13%
Current study, 2023	R	175	CEC	CF/K	60 Gy	41.6 mo	2y LRFS 52%	2y OS 59%, PFS 47%	Grade ≥ II 29.7%

Abbreviations: C: cisplatin, CEC: cervical esophageal carcinoma, CF: cisplatin, fluorouracile, Grade: grade, K: carboplatin, LC: local control, LFFS: local failure-free survival, LRPFS: locoregional progession-free survival, mo: months, n: patient number, OS: overall survival, P: prospective, PFS: progression free survival, R: retrospective, TEC: thoracic esophageal carcinoma, Tx: toxicity.

In the definitive treatment of CEC, concurrent chemotherapy typically consists of platin-based regimens, particularly cisplatin and 5-FU, oxaliplatin and 5-FU, or carboplatin and paclitaxel [4]. Bleiberg et al. [25] compared cisplatin alone (100 mg/m^2^) to cisplatin and continuous 5-FU infusion in EC patients, reporting improved two-year OS and increased toxicity rates in the combined treatment group. Other chemotherapeutic regimens, such as FOLFOX or carboplatin and pactitaxel-based agents, have been studied and found to be as effective as cisplatin and 5-FU [26,27]. Finally, in the SCOPE1 trial, the role of cetuximab as an epidermal growth factor receptor-targeting agent was investigated, but it was not recommended due to treatment-limiting toxicity [28]. We found no statistically significant difference in outcomes or toxicity when comparing concurrent chemotherapy regimens in our study, as was previously reported.

Patients with CEC who undergo CRT have a better chance of survival if their primary tumor can be controlled locally. Some authors, concerned about the poor prognoses of CRT non-responders, have advocated for more drastic measures, such as early salvage surgery [29]. Zhang et al. [21] suggested that superior LC was associated with a lower incidence of distant metastasis. Similarly, Uno et al. [30] found that the LC of the primary tumor is directly related to survival. Lastly, Zenda et al. [6] reported a three-year OS for patients with CR of 74.6%, compared to 25.0% for patients without CR (*p* = 0.002). Patients with CR had significantly better survival and locoregional control than non-responders, according to our findings.

There are some limitations to our study. The study can only be considered retrospective, which inherently presents selection bias. Further complicating the ability to draw definitive conclusions is the fact that this multicentric study employed a wide range of chemotherapy agents and RT doses. Finally, appreciation of overall toxicity may be confounded because the toxicity analysis was performed retrospectively based on patient charts. Our study also has some strengths. Although many studies have investigated the use of CRT for CEC patients, our study is unique because it only used studies with large cohorts and which investigated only patients with SCC histology and definitive treatment protocols. This allowed us to focus on dose escalation in such patients in the absence of prospective trials. Additionally, we believe that the vast majority of the patients in the studies included here were staged with PET-CT and treated with IMRT, reflecting current treatment modalities.

## Conclusion

Our findings show that CRT is a safe and effective treatment option for patients with CEC. We found no benefit to increasing RT dose beyond 54 Gy in the presence of chemotherapy, and higher doses were associated with increased toxicity. Local control of the primary tumor is critical for survival. Prospective studies with larger cohorts are needed to validate our findings.

## Data Availability

Research data are stored in an institutional repository and will be shared upon request to the corresponding author.
